# Characterization and Mapping of Spot Blotch in *Triticum durum–Aegilops speltoides* Introgression Lines Using SNP Markers

**DOI:** 10.3389/fpls.2021.650400

**Published:** 2021-05-28

**Authors:** Jashanpreet Kaur, Jaspal Kaur, Guriqbal Singh Dhillon, Harmandeep Kaur, Jasvir Singh, Ritu Bala, Puja Srivastava, Satinder Kaur, Achla Sharma, Parveen Chhuneja

**Affiliations:** ^1^Department of Plant Pathology, Punjab Agricultural University, Ludhiana, India; ^2^Department of Plant Breeding and Genetics, Punjab Agricultural University, Ludhiana, India; ^3^School of Agricultural Biotechnology, Punjab Agricultural University, Ludhiana, India

**Keywords:** leaf blight, spot blotch, backcross introgression lines, *Aegilops speltoides*, *Bipolaris sorokiniana*, *Triticum durum*, QTL

## Abstract

Spot blotch (SB) of wheat is emerging as a major threat to successful wheat production in warm and humid areas of the world. SB, also called leaf blight, is caused by *Bipolaris sorokiniana*, and is responsible for high yield losses in Eastern Gangetic Plains Zone in India. More recently, SB is extending gradually toward cooler, traditional wheat-growing North-Western part of the country which is a major contributor to the national cereal basket. Deployment of resistant cultivars is considered as the most economical and ecologically sound measure to avoid losses due to this disease. In the present study, 89 backcross introgression lines (DSBILs) derived from *Triticum durum* (cv. PDW274-susceptible) × *Aegilops speltoides* (resistant) were evaluated against SB for four consecutive years, 2016–2020. Phenotypic evaluation of these lines showed a continuous variation in disease severity indicating that the resistance to SB is certainly quantitative in nature. Phenotypic data of DSBILs were further used for mapping QTLs using SNPs obtained by genotyping by sequencing. To identify QTLs stable across the environments, Best Linear Unbiased Estimates (BLUEs) and Predictions (BLUPs) were used for mapping QTLs based on stepwise regression-based Likelihood Ratio Test (RSTEP-LRT) for additive effect of markers and single marker analysis (SMA). Five QTLs, *Q.Sb.pau-2A*, *Q.Sb.pau-2B*, *Q.Sb.pau-3B*, *Q.Sb.pau-5B*, and *Q.Sb.pau-6A*, linked to SB resistance were mapped across chromosomes 2A, 2B, 3B, 5B, and 6A. Genes found adjacent to the SNP markers linked to these QTLs were literature mined to identify possible candidate genes by studying their role in plant pathogenesis. Further, highly resistant DSBIL (DSBIL-13) was selected to cross with a susceptible hexaploidy cultivar (HD3086) generating BC_2_F_1_ population. The QTL *Q.Sb.pau-5B*, linked to SNP S5B_703858864, was validated on this BC_2_F_1_ population and thus, may prove to be a potential diagnostic marker for SB resistance.

## Introduction

Wheat, a major food crop of the world population, is in a constant threat from various biotic and abiotic stresses, limiting its potential for yield. Helminthosporium leaf blight/foliar blight/spot blotch (SB), caused by *Cochliobolus sativus* (anamorph: *Bipolaris sorokiniana*), is a major foliar disease of wheat in warmer wheat-growing regions. This hemibiotrophic fungus can potentially infect and damage various species of Poaceae family (Gupta et al., 2018). Due to drastic changes in the weather conditions in the last few decades leading to higher average temperature and unusual rainfall patterns, foliar leaf blight is emerging as a major threat to wheat production in India. Globally, an estimated 25 million ha of wheat land is affected by SB (Yadav et al., 2015), out of which around 10 million ha is in the Indian Subcontinent and 9 million ha of this is in the North-Eastern Plain Zone of India (Duveiller and Sharma, 2012; Chowdhury et al., 2013). This disease is extending gradually toward the North-West part characterized by high temperature and humidity late in the season (Saari, 1998) with an average yield loss of about 15–20% (Chand et al., 2003). The disease also causes serious damage in seed quality and market value of the produce leading to substantial economic losses (Singh and Kumar, 2008). Under heavy infestation, yield losses vary from 80 to 100% (Kumar et al., 2008). It is chiefly a seed-transmitted disease and the conidia can also survive in the soil.

Considering the huge wheat acreage attacked by this disease, it becomes obligatory to tackle this disease in wheat-growing areas through use of disease-free seed, seed treatment with a suitable fungicide reducing the carryover inoculum, and crop rotation to provide enough window period for decomposition of inoculum-carrying stubble (Chowdhury et al., 2013). Fungicide application seems to be the most convenient method. However, their repeated application involves significant cost, health hazard, and emergence of fungicidal resistance in the target pathogen. Among various alternatives, deployment of resistant cultivars remains a top priority approach as genetic resistance is an economical, robust, and environmentally friendly tool in the management of leaf blight disease. Resistance to leaf blight in the commonly grown wheat varieties of South-East Asia is generally insufficient or lacking (Joshi et al., 2004). So, there is an urgent need to identify sources of SB resistance from the gene pool of wild relatives.

From the limited number of inheritance studies, it has been found that both qualitative and quantitative type of inheritance are involved in SB resistance. A number of bi-parental studies and association mapping studies have reported QTLs linked to SB resistance present all over the wheat genome. Among them, four major QTLs, *Sb1* on 7D (Lillemo et al., 2013), *Sb2* on 5B (Kumar et al., 2015), *Sb3* on 3B (Lu et al., 2016), and *Sb4* on 4B (Zhang et al., 2020), have been identified and mapped. Several QTLs on chromosomes 2AL, 2BS, 5BL, and 6DL in “Yangmai#6”; on 2AS, 2BS, 5BL, and 7DS in the cultivar “Ning#8201”; and on 2BS, 2DS, 3BS, 7BS, and 7DS in the cultivar “Chirya#3” have been reported (Kumar et al., 2008, 2010). Neupane et al. (2010) reported a single, dominant gene conditioned resistance to leaf blight in “Chirya#3” and “Milan/Sanghai#7.” Association mapping studies conducted by Gurung et al. (2014) and Adhikari et al. (2012) identified genomic regions associated with resistance to SB on chromosomes 1A, 1B, 3B, 5B, 6B, 7B, and 7D.

However, identification of donor lines resistant to SB remains a major continuing challenge (Joshi et al., 2007). At CIMMYT, a number of *Aegilops* and *Triticum* species were used as donors for resistance to leaf blight which included *Aegilops triuncialis*, *Aegilops cylindrica*, *Aegilops speltoides*, *Aegilops triaristata*, *Triticum dicoccoides* (wild emmer wheat), *Triticum boeoticum*, *Triticum persicum*, *Triticum timopheevii*, *Triticum araraticum*, *Triticum urartu*, and *Triticum sphaerococcum* (Singh and Dhaliwal, 1993; Smurova and Mikhailova, 2007). *Aegilops* species is considered as a good and less exploited source for increasing the genetic potential of cultivated wheat to various biotic and abiotic stresses.

In the wide hybridization program at Punjab Agricultural University, Ludhiana, a set of stable interspecific backcross introgression lines derived from *Triticum durum* and *A. speltoides* (DSBILs), putative B genome donor of wheat, were evaluated under polyhouse conditions for four consecutive seasons from 2016 to 2020 against SB. These DSBILs were used further to detect QTL(s) governing SB resistance and identify linked markers to aid in breeding for disease resistance in wheat. The linked markers were further used for validation on a BC_2_F_1_ population derived from HD3086 and one of the resistant DSBILs.

## Materials and Methods

### Plant Genetic Material

A total of 89 backcross introgression lines derived from *A. speltoides* (accession #pau3809) and *T. durum* cultivar “PDW274” as recurrent parent were screened for resistance against SB. The F_1_ plants from the cross of *T. durum* cv. PDW274 and *A. speltoides* acc. pau3809 were backcrossed for two generations with *T. durum* and selfed to generate BC_2_F_10_ introgression lines (DSBILs). Details of development of material can be retrieved from Awlachew et al. (2016).

### Screening for SB Resistance

All the 89 DSBILs along with resistant parent *A. speltoides*, recurrent parent PDW274, and hexaploid susceptible check “Raj 4015” were evaluated under polyhouse conditions following artificially induced epiphytotic conditions. Susceptible check WL711 was sown after every 20 rows, and also in alleys to promote inoculum build-up and spread. Screening to leaf blight disease was done during four consecutive wheat seasons 2016–2017 (E1), 2017–2018 (E2), 2018–2019 (E3), and 2019–2020 (E4). Artificial epiphytotic conditions were created by spraying conidial suspension of the pathogen *B. sorokiniana* maintained on sorghum grains which were previously soaked and autoclaved. Aqueous conidial suspension (10^6^ conidia/ml) was sprayed on plants during evening hours until symptoms appeared on the susceptible checks. After inoculation, plants were lightly irrigated to provide high-humidity conditions, which is one of the predisposing conditions for infection by *B. sorokiniana*. Disease scoring was done at three different growth stages (GS) on Zadoks’ scale (Zadoks et al., 1974), which are GS55 (flowering stage or FS), GS75 (medium milk/dough stage or DS), and GS87 (hard dough stage or HDS), using a double-digit scale (00–99) which is based on percent leaf area covered due to blight in flag leaf and one leaf below flag leaf (F-1) as mentioned in Supplementary Table 1. The digit toward the left side indicates score of percent blighted area on flag leaf whereas the right digit gives the score of penultimate/F-1 leaf (Khan and Chowdhury, 2011). These two leaves at this stage contribute most to the grain-filling process thus directly affecting the grain yield (Chowdhury et al., 2013).

The AUDPC (area under disease progress curve) based on disease severity at GS55 (FS), GS75 (DS), and GS87 (HDS) was calculated as the total area under the graph of disease severity against time *t*, from the first disease evaluation to the last, with the following equation as given by Shaner and Finney (1977):

$$A\,U\,D\,P\,C=S_{i}=\sum_{i}^{n-1}\left[\left(t_{i-1}-t_{i}\right)\,\left(y_{i}+y_{i+1}\right)/2\right]$$

where *y*_*i*_ = disease severity at time, (*t*_*i*__+__1_–*t*_*i*_) = time in days between two disease scores, and *n* = number of dates for which SB disease level was recorded.

### Statistical Analysis

The disease severity scores across different years using scores of FS, DS, HDS, and AUDPC were used to obtain best linear unbiased estimates (BLUEs) and predictions (BLUPs) by fitting linear mixed effects models in lme4 package v 1.1-26 (Bates et al., 2015) in R v4.0.3 (R Core Team, 2019) using

$$Y_{i\,k}=\mu +Y\,e\,a\,r_{i}+L\,i\,n\,e_{k}+\varepsilon_{i\,k}$$

where *Y*_*ik*_ is the trait of interest, μ is the mean effect, *Year*_*i*_ is the effect of the *i*th year, *Line*_*k*_ is the effect of the *k*th line, and ε_*ik*_ is the error associated with the *i*th year and the *k*th line, which is assumed to be normally and independently distributed, with mean zero and homoscedastic variance σ^2^. For BLUEs model, the genotypes were considered as fixed effects, while for BLUPs model all the effects were considered as random effects. Considering genotypes as random effects reduces the effect of screening time along with other environmental effects on SB severity (Tomar et al., 2021). The disease severity scores obtained by fitting the BLUPs and BLUEs models were plotted using ggplot2 v3.3.3 (Wickham, 2016) and ggpubr v0.4.0 (Kassambara, 2020) in R v4.0.3 to study the distribution across the DSBILs.

Further, principal component analysis was performed to identify the number of principal components required to explain the variation across the years along with fitted values from linear mixed effect models using FactoMineR v2.4 (Lê et al., 2008) and factoextra v1.0.7 (Kassambara and Mundt, 2020) in R v4.0.3. The principal components were plotted as biplots to study the relation between disease severity scores of FS, DS, HDS, and AUDPC along with identification of reduction in environmental effects in fitted values.

### Genotyping

DNA extraction for all the 89 DSBILs along with both the parents was done using modified cetyltrimethylammonium bromide (CTAB) method (Saghai-Maroof et al., 1984). All these DNA samples were genotyped with genotyping-by-sequencing (GBS) to provide dense genome-wide marker coverage. Raw sequence files were processed in the TASSEL GBS pipeline version 5.2.31 (Glaubitz et al., 2014) and further aligned to the International Wheat Genome Sequencing Consortium (IWGSC) RefSeq v1.0 reference genome. The vcf file so obtained was filtered for a minimum depth at 3 (DP3) and converted to hapmap format. The TASSEL output was then filtered for homozygous SNPs for each parental line and SNP markers polymorphic between the two parents were selected, and loci with very low coverage (<50%)/high missing data (>50%) or heterozygosity (>50%) were filtered out. DSBILs with more that 10% missing data were filtered out. Missing SNPs were imputed using the LD-kNNi method implemented in TASSEL with the following default parameters of minimum number of high LD sites = 30 and number of nearest neighbors = 10 (Ladejobi et al., 2019). SNPs with minor allele frequency (MAF) < 0.05 were excluded from further analysis and finally, 4056 SNPs with good quality genotype calls for 77 DSBILs along with recurrent parent were used for mapping.

### QTL Mapping Using QTL IciMapping

QTL mapping was done by using CSL functionality of QTL IciMapping version 4.1 software (Meng et al., 2015). Disease resistance mapping was conducted with 4056 SNPs (MAF > 0.05) in 77 DS-BILs plus recurrent parent by stepwise regression-based Likelihood Ratio Test (RSTEP-LRT) for additive effect of markers and single marker analysis (SMA) in the software. Stepwise regression was used to determine the percentages of phenotypic variance explained (PVE) (R^2^) by individual QTL and their respective additive effects at the likelihood of odds ratio (LOD) peaks. Significant SNPs were identified using threshold LOD of 3 at significant *p* ≤ 0.001 and 1000 permutations. Only QTLs detected by both the algorithms and using both BLUPs and BLUEs were considered stable and significant. Further, the allelic effects were investigated to identify significantly associated markers with phenotypic data by Kruskal–Wallis test for studying the importance of individual alleles in SB disease resistance.

### Postulation of Candidate Genes

The identified QTLs were further used to identify genic regions adjacent to their linked SNPs using reference genome assembly’s functional annotation for high confidence genes (IWGSC Ref Seq v1.0). The genes were retrieved from a region of 500 kb on either side of the SNP and using the functional annotations, the proteins coded by these genes were identified. The functions of the proteins were further literature mined to identify their role in imparting resistance against SB.

### Validation of the Identified QTLs and Markers

For validation of the identified QTLs and markers, a BC_2_F_1_ population was developed from bread wheat cv. ‘‘HD3086’’ (high yielding, susceptible cultivar) and one of the DSBILs showing highly resistant response persistently under polyhouse conditions. All the plants were evaluated by creating artificial epiphytotic conditions as explained previously and scoring was done using a double-digit scale. Genomic DNA for all plants of BC_2_F_1_ mapping population and parents was extracted using CTAB method. To validate the SNP markers significantly linked to SB resistance as identified in mapping results, Kompetitive allele-specific PCR (KASP) assay was used for genotyping^1^. For this purpose, SNPs linked to the QTLs were used to design KASP markers.

## Results

### Phenotypic Evaluation of DSBILs

**FIGURE 1:**
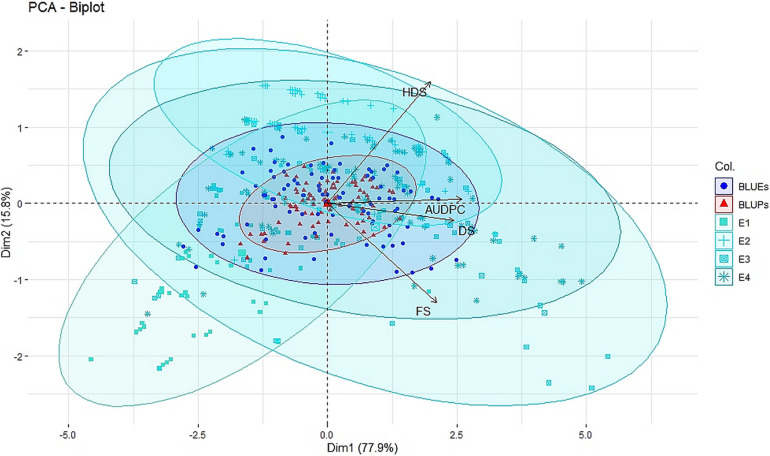
Principal component analysis (PCA) biplot of disease severity of spot blotch across four individual environments E1, E2, E3, and E4 (turquoise color), across environment BLUEs (blue color), and across environment BLUPs (red color) for disease severity at flowering stage (FS), dough stage (DS), hard dough stage (HDS), and AUDPCs.

**FIGURE 2:**
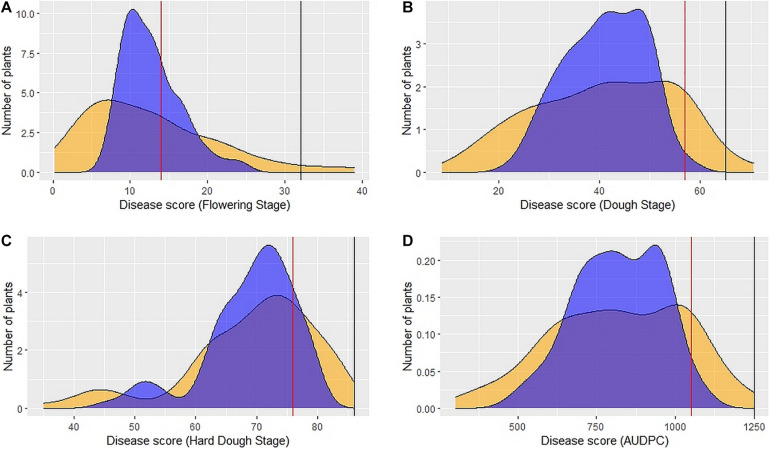
Distribution of 89 DSBILs for spot blotch severity based on BLUEs (yellow color) and BLUPs (blue color) for disease score at flowering stage **(A)**, dough stage **(B)**, hard dough stage **(C)** and AUDPC **(D)**. The vertical red line indicates disease score of recurrent parent PDW274, the vertical black line indicates disease score of susceptible check Raj4015, and the disease score or *A. speltoides* was 00 across the stages.

Large variation in disease severity was observed across the different growth stages with disease pressure increasing from flowering to hard dough stage (Table 1 and Supplementary Table 2). Across the environments, overall disease pressures were the lowest in E1 and highest in E3. To enhance the accuracy and map stable QTLs across the environments, linear mixed-effects models were used to obtain fitted values of disease severity, accounting for G × E effect. These values are termed as BLUPs (genotypes as random effects) and BLUEs (genotypes as fixed effects) from here onward. Plotting the eigenvalues/variances explained by each individual principal component (from PC1 to PC2), across the different growth stages and AUDPCs for all the environments, including BLUEs and BLUPs, showed that the first two principal components explained 93.7% of total (Supplementary Figure 1). The first two dimensions of principal components showed both BLUEs and BLUPs were able to explain the variance of disease scores across the four environments (Figure 1). The BLUPs showed lower variance than the BLUEs which meant BLUPs were able to reduce the environmental variance across the years to a larger extent. The disease score distribution curves further agreed to this showing better normal distribution (Figure 2).

**TABLE 1:** Phenotypic evaluation for spot blotch disease severity of DSBILs along with recurrent parent (RP) and susceptible check.

**Env**	**Stage**	**RP**	**Check**	**Population**						
		**PDW274**	**Raj4015**	**Range**	**Median**	**Mean**	**SD**	**CV**	**Skew.**	**Kurt.**
BLUEs	FS	14.50	38.00	00.25–39.00	11.25	12.71	8.42	0.66	0.96	0.54
	DS	62.00	70.25	08.75–70.50	42.50	41.02	13.83	0.34	−0.20	−0.86
	HDS	77.75	89.00	35.00–83.75	72.25	69.17	10.76	0.16	−1.07	0.93
	AUDPC	1081.25	1337.50	302.50–1228.75	828.75	819.55	213.24	0.26	−0.23	−0.73
BLUPs	FS	13.61	24.20	07.18–24.65	12.14	12.81	3.79	0.30	0.96	0.56
	DS	52.92	57.58	22.82–57.73	41.90	41.09	7.76	0.19	−0.21	−0.83
	HDS	75.00	82.81	45.32–79.17	71.18	69.06	7.43	0.11	−1.09	1.01
	AUDPC	985.25	1147.62	491.81–1078.71	825.26	819.86	134.52	0.16	−0.24	−0.70

*RP, recurrent parent; CV, coefficient of variation; Skew., skewness; Kurt., kurtosis; Env, environment; BLUEs, best linear unbiased estimates; BLUPs, best linear unbiased predictions; FS, flowering stage; DS, dough stage; HDS, hard dough stage; AUDPC, area under disease progression curve.*

*Donor parent *Aegilops speltoides* (#pau3809) showed score 00 across all stages.*

The donor parent *A. speltoides* acc. pau3809 was found to be immune to SB showing highly resistant disease severity score of 00, on the double-digit scale, across all the growth stages studied. Overall, the recurrent parent PDW274 showed moderate to high susceptibility and the susceptible check Raj4015 showed high susceptibility across the growth stages and AUDPCs when compared with range of disease scores of respective data sets. At FS, disease score BLUEs of the recurrent parent PDW274 and susceptible check Raj4015 were 14.5 and 38.0, respectively, while for the DSBILs, it ranged from 0.25 to 39.00. The disease score BLUPs of PDW274 and Raj4015 were 13.61, and 24.20, respectively with DSBILs showing a range from 07.18 to 24.65 (Table 1). At DS, disease score BLUEs of the recurrent parent PDW274 and susceptible check Raj4015 were 62.00 and 70.25, respectively, while for the DSBILs, it ranged from 08.75 to 70.50. The disease score BLUPs of PDW274 and Raj4015 were 52.92 and 57.58, respectively, with DSBILs showing a range from 22.82 to 57.73. At HDS, disease score BLUEs of the recurrent parent PDW274 and susceptible check Raj4015 were 77.75 and 89.00, respectively, while for the DSBILs, it ranged from 35.00 to 83.75. The disease score BLUPs of PDW274 and Raj4015 were 75.00 and 82.81, respectively, with DSBILs showing a range from 45.32 to 79.17.

The AUDPC values showed a similar trend, where the disease score BLUEs of the recurrent parent PDW274 and susceptible check Raj4015 were 1081.25 and 1337.50, respectively, while for the DSBILs, it ranged from 302.50 to 1228.75. The disease score BLUPs of PDW274 and Raj4015 were 985.25 and 1147.62, respectively, with DSBILs showing a range from 491.81 to 1078.71. Only three genotypes, DS13, DS61, and DS80, were found highly resistant across all the growth stages. Overall, less than 1% of lines were categorized under highly resistant category while 29 and 25% of genotypes showed moderate to high susceptibility, respectively. The rest of the lines fell under resistant to moderately resistant category.

### QTL Mapping

**FIGURE 3:**
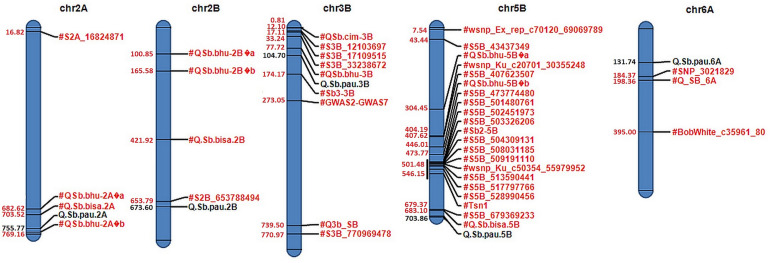
Physical map of candidate QTLs on 2A, 2B, 3B, 5B, and 6A chromosomes. Significant QTLs mapped for resistance against spot blotch are highlighted in black with their respective physical positions (in Mb) in blue, while previously reported QTLs/markers are labeled in red.

QTL mapping using both SMA and RSTEP-LRT for additive effect of markers using BLUPs and BLUEs for disease severity scores at different GS and AUDPCs resulted in detection of five QTLs across five chromosomes (Table 2 and Figure 3). These QTLs were located on chromosomes 2A, 2B, 3B, 5B, and 6A. The phenotypic variation explained by these QTLs varied from 16.03 to 25.56%, while the LOD score varied from 3.04 to 5.02. QTL *QSb.pau-2A* was mapped at chromosome 2A at 755.77 Mb using disease severity at hard dough stage with LOD 3.12, PVE 18.44% using BLUEs and LOD 3.18, PVE 18.77% using BLUPs with resistance allele contributed by *A. speltoides*. Two QTLs, *Q.Sb.pau-2B* and *Q.Sb.pau-3B*, were mapped using both disease severity at HDS and AUDPC, where the resistant allele for *Q.Sb.pau-2B* was contributed by PDW274 while the resistant allele for *Q.Sb.pau-3B* was contributed by *A. speltoides* in both cases. QTL *QSb.pau-2B* was mapped at chromosome 2B at 673.60 Mb using disease severity at HDS with LOD 4.09, PVE 21.27% using BLUEs and LOD 5.02, PVE 25.56% using BLUPs. Using AUDPCs, it was mapped with LOD 3.04, PVE 16.03% using BLUEs and LOD 3.16, PVE 16.98% using BLUPs. QTL *QSb.pau-3B* was mapped at chromosome 3B at 104.70 Mb using disease severity at HDS with LOD 3.22, PVE 17.86% using BLUEs and LOD 3.82, PVE 20.43% using BLUPs. Using AUDPCs, it was mapped with LOD 4.33, PVE 23.27% using BLUEs and LOD 4.58, PVE 25.27% using BLUPs.

**TABLE 2:** Summary of the QTLs detected using both single marker analysis (SMA) and RSTEP-LRT for additive effect of markers algorithms of QTL ICI mapping for spot blotch disease severity.

**QTL**	**Marker**	**Chr**	**Pos (Mb)**	**GS**	**Env**	**LOD**	**PVE (%)**	**Add**
*Q.Sb.pau-2A*	S2A_755774702	2A	755.77	HDS	BLUEs	3.12	18.44	−8.24
					BLUPs	3.18	18.77	−5.73
*Q.Sb.pau-2B*	S2B_673595704	2B	673.60	AUDPC	BLUEs	3.04	16.03	160.96
					BLUPs	3.16	16.98	149.57
				HDS	BLUEs	4.09	21.27	11.81
					BLUPs	5.02	25.56	10.35
*Q.Sb.pau-3B*	S3B_104700872	3B	104.70	AUDPC	BLUEs	4.33	23.27	−128.66
					BLUPs	4.58	25.27	−113.13
				HDS	BLUEs	3.22	17.86	−8.62
					BLUPs	3.82	20.43	−7.79
*Q.Sb.pau-5B*	S5B_703858864	5B	703.86	DS	BLUEs	3.32	19.48	−6.06
					BLUPs	3.23	18.97	−3.36
*Q.Sb.pau-6A*	S6A_131743987	6A	131.74	FS	BLUEs	3.07	16.38	7.59
					BLUPs	3.08	16.42	3.42

*Chr, chromosome; Pos Mb, position in million bases; GS, growth stage; Env, environment; LOD, logarithm of odds; PVE, phenotypic variation explained; Add, additive effect; BLUEs, best linear unbiased estimates; BLUPs, best linear unbiased predictions; FS, flowering stage; DS, dough stage; HDS, hard dough stage; AUDPC, area under disease progression curve.*

Only one QTL was mapped for disease severity at FS and DS. QTL *QSb.pau-5B* was mapped at chromosome 5B at 703.86 Mb using disease severity at DS with LOD 3.32, PVE 19.48% using BLUEs and LOD 3.23, PVE 18.97% using BLUPs with resistance allele contributed by *A. speltoides*. QTL *QSb.pau-6A* was mapped at chr6A at 131.74 Mb using disease severity at FS with LOD 3.07, PVE 16.38% using BLUEs and LOD 3.08, PVE 16.42% using BLUPs with resistance allele contributed by PBW274.

### Allelic Effect of Identified QTLs

**FIGURE 4:**
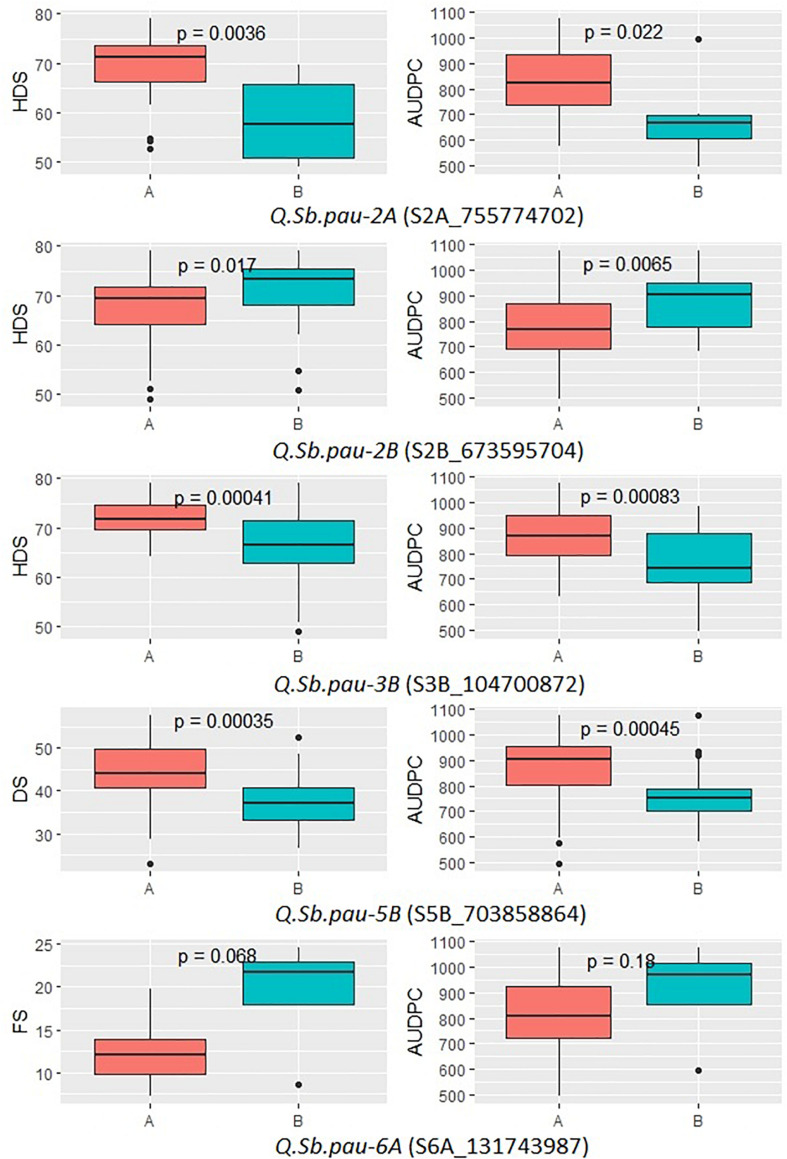
Boxplots showing the effect of phenotypic variation between the two alleles of the SNP markers linked to QTLs for disease score of DSBILs. Kruskal–Wallis test was used to determine the significant differences between the mean values of two alleles.

The allelic effect of the SNPs linked to SB QTL was plotted for the five significant QTLs (Figure 4). The allelic variation patterns of the QTLs, between the two alternate alleles, further agreed with positive mapping results. Also, the donor parent of resistant alleles was confirmed for the QTLs. The disease severity scores of growth stages in which the QTLs were detected were used along with AUPDCs. The patterns of disease severity for the alternate alleles confirmed that three of the QTLs (*QSb.pau-2A*, *QSb.pau-3B*, and *QSb.pau-5B*) had resistant allele donated by *A. speltoides*, while the remaining two QTLs (*QSb.pau-2B*, and *QSb.pau-6A*) had resistant allele donated by PDW274. The alternate alleles of QTL *QSb.pau-3B* and *QSb.pau-5B* were the most significantly different for respective disease severity score and AUDPCs, while the alternate alleles of QTL *QSb.pau-6A* were least significantly different.

### Postulation of Candidate Genes

Because the physical locations of the SNPs linked to the QTLs detected in the present study were known, they were used to identify the genes present adjacent to them in a region of 500 kb on either side of the SNP (Supplementary Table 3). For each target locus, the regions were inspected to identify candidate genes for the QTL and the genes known to be involved in different pathways of pathogen–host interactions and pathogenesis were considered to understand their role in imparting resistance to SB (Table 3). The SNP S2A_755774702 linked to QTL *QSb.pau-2A* was found adjacent to genes *TraesCS2A01G547800* and *TraesCS2A01G547900*. The gene *TraesCS2A01G547800* codes for Auxin response factor (ARF) and *TraesCS2A01G547900* codes for Zinc finger CCCH domain-containing protein 32. Gene *TraesCS2A01G547400* was found in close vicinity of the QTL for FBD, F-box, and Leucine Rich Repeat domains protein. Other genes in the genomic region with probable role in disease resistance coded for cysteine proteinase (*TraesCS2A01G546700*), Cytochrome P450 (*TraesCS2A01G546600* and *TraesCS2A01G547600*), and Zinc finger MYM-type-like protein (*TraesCS2A01G546800*). The SNP S2B_673595704 linked to QTL *QSb.pau-2B* was found adjacent to genes *TraesCS2B01G476500* and *TraesCS2B01G476600* both encoding senescence-associated family protein (DUF581). Four other genes coding for DUF581 were also found in the genomic region of the QTL. The SNP S3B_104700839 linked to QTL *Q.Sb.pau-3B* was found adjacent to genes *TraesCS3B01G127000* and *TraesCS3B01G127100*. The gene *TraesCS3B01G127000* coded for protein FAR1-RELATED SEQUENCE 3 and gene *TraesCS3B01G127000* coded for IQ domain-containing protein.

**TABLE 3:** QTLs along with SNPs and corresponding proteins and functional gene annotation elucidated based on the high confidence genes from wheat reference sequence (RefSeq V1.0) annotation database.

**QTL**	**SNP**	**Chr**	**GeneID**	**Dist. (kb)**	**Gene annotation**
*Q.Sb.pau-2A*	S2A_755774702	2A	*TraesCS2A01G546600*	439.059	Cytochrome P450 family protein, expressed
			*TraesCS2A01G546700*	435.192	Cysteine proteinase
			*TraesCS2A01G546800*	418.040	Zinc finger MYM-type-like protein
			*TraesCS2A01G547400*	81.978	FBD, F-box and Leucine Rich Repeat domains protein
			*TraesCS2A01G547600*	37.854	Cytochrome P450, putative
			*TraesCS2A01G547800*	5.678	Auxin response factor
			*TraesCS2A01G547900*	−11.024	Zinc finger CCCH domain-containing protein 32
*Q.Sb.pau-2B*	S2B_673595704	2B	*TraesCS2B01G476400*	425.139	Senescence-associated family protein (DUF581)
			*TraesCS2B01G476500*	353.761	Senescence-associated family protein (DUF581)
			*TraesCS2B01G476600*	−32.658	Senescence-associated family protein (DUF581)
			*TraesCS2B01G476700*	−112.168	Senescence-associated family protein (DUF581)
			*TraesCS2B01G476800*	-115.776	Senescence-associated family protein (DUF581)
			*TraesCS2B01G476900*	−329.070	Senescence-associated family protein (DUF581)
*Q.Sb.pau-3B*	S3B_104700839	3B	*TraesCS3B01G127000*	375.807	Protein FAR1-RELATED SEQUENCE 3
			*TraesCS3B01G127100*	−269.633	IQ domain-containing protein
*Q.Sb.pau-5B*	S5B_703858864	5B	*TraesCS5B01G553200*	370.394	F-box family protein
			*TraesCS5B01G553300*	361.335	F-box domain containing protein
			*TraesCS5B01G553400*	358.507	F-box and associated interaction domains protein
			*TraesCS5B01G553500*	329.318	F-box domain containing protein, expressed
			*TraesCS5B01G553700*	214.322	F-box family protein
			*TraesCS5B01G553900*	1.689	F-box family protein
			*TraesCS5B01G554000*	−214.708	ATP-dependent Clp protease ATP-binding subunit
			*TraesCS5B01G554100*	−232.540	F-box family protein
			*TraesCS5B01G554200*	−250.728	Disease resistance protein RPM1
			*TraesCS5B01G554300*	−276.340	Disease resistance protein (NBS-LRR class) family
			*TraesCS5B01G554500*	−368.461	AIG2-like (Avirulence induced gene) family protein
*Q.Sb.pau-6A*	S6A_131743987	6A	*TraesCS6A01G149500*	297.870	Ubiquitin family protein
			*TraesCS6A01G149600*	−436.076	Uricase

*Distance from SNP (Dist.) represents distance of start site of gene to SNP linked with QTL, where (+) sign indicates that the gene was found downstream of the SNP and (−) sign indicates that the gene was found upstream.*

The SNP S5B_703858864 linked to QTL *Q.Sb.pau-5B* was found adjacent to genes *TraesCS5B01G553900* and *TraesCS5B01G554000*. The gene *TraesCS5B01G553900* coded for F-box family protein and gene *TraesCS5B01G554000* coded for ATP-dependent Clp protease ATP-binding subunit. Along with six other F-box family protein coding genes in the region, three disease resistance protein genes RPM1 (*TraesCS5B01G554100*), NBS-LRR family protein (*TraesCS5B01G554200*), and AIG2 like protein (*TraesCS5B01G554300*) were found in the genomic region of the QTL. The SNP S6A_131743987 linked to QTL *Q.Sb.pau-6A* was found adjacent to genes *TraesCS6A01G149500* and *TraesCS6A01G149600*. The gene *TraesCS6A01G149500* coded for Ubiquitin family protein and gene *TraesCS6A01G149600* coded for uricase.

### Validation of the Identified QTLs and Markers

BC_2_F_1_ population derived from DSBIL13 × HD3086 was generated for transfer of SB resistance into wheat and to validate the identified SNP markers linked to SB QTLs, where HD3086 is a high-yielding SB-susceptible hexaploid cultivar and DSBIL13 is a highly resistant line. Besides being highly resistant to SB, DSBIL13 also harbored resistant alleles of four out of five QTLs mapped in the present study, namely *QSb.pau-2A*, *QSb.pau-2B*, *QSb.pau-3B*, and *QSb.pau-5B*. About 75% of the plants were found to show resistance reaction when screened phenotypically under polyhouse conditions.

**FIGURE 5:**
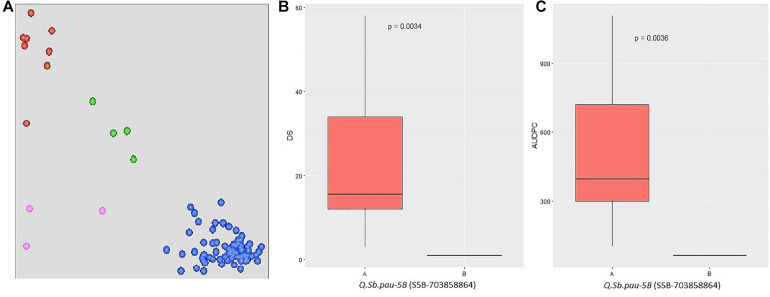
**(A)** Validation of QTL using KASP assay on BC_2_F_1_ population from DS13/HD 3086 with marker S5B_703858864. The central cluster (green) represents heterozygous individuals, whereas clusters near the axes are homozygous for resistant allele (HEX; red) and susceptible allele (FAM; blue). **(B,C)** Boxplots showing the effect of phenotypic variation between the two alleles of the QTLs for disease score and AUDPC of BC_2_F_1_ population. Kruskal–Wallis test was used to determine the significant differences between the mean values of two alleles.

The SNPs linked to the five SB resistance QTLs were converted to KASP markers (Supplementary Table 4) and parental polymorphism survey was done to study the allelic composition of HD3086, DSBIL13, PDW274, and *A. speltoides* acc. pau3809. Out of five markers, only S5B_703858864 was found to be polymorphic between HD3086 and DSBIL13, i.e., HD3086 harbored an alternate allele to the allele imparting resistance. Thus, only this marker could be successfully used to track the SB resistance allele of QTL *QSb.pau-5B*. This marker was then applied to BC_2_F_1_ population derived from DSBIL13 × HD3086. The disease severity scores of growth stages in which the QTL was detected was used along with AUPDCs to evaluate significance of differences by Kruskal test of significant difference (Figure 5). The patterns of disease severity for the alternate alleles confirmed that the QTL *QSb.pau-5B* having resistant allele from *A. speltoides* was transferred to the BC_2_F_1_ population with significant difference of alternate alleles at *p* = 0.0034 for DS and *p* = 0.0036 for AUDPC. Thus, this marker can be used for marker-assisted selection (MAS) and gene pyramiding in future crop improvement programs.

## Discussion

Spot blotch is one of the major constraints to the global wheat production, especially in areas with hot and humid climate (Tomar et al., 2021). To counter the constraints from foliar diseases like SB, there is a need for constantly identifying and introgressing new sources of resistance. The DSBIL panel used in the present study showed wide range of variation for different traits and has already been reported to possess various QTLs for heat tolerance, stripe rust, and powdery mildew resistance (Awlachew et al., 2016; Dhillon et al., 2020). In the present study, during phenotypic evaluation of disease severity for SB, three DSBILs (DSBIL 13, 61, and 80) were identified to be highly resistant against SB. Because no wheat cultivar presently grown in North-Western plains of India possess resistance to SB, these lines become an important resource for transfer of SB resistance. The phenotypic evaluation showed increased disease severity from FS (GS55) to HDS (GD87). At HDS, immunity was mostly characterized by moderate resistance in the DSBIL panel. The continuous distribution of disease severity score in the panel indicated additive effect leading to quantitative nature of resistance. Most of the studies on SB resistance dictate that multiple genes with minor effect control the SB resistance in wheat (Adhikari et al., 2012; Lillemo et al., 2013; Zhuang et al., 2013; Gurung et al., 2014; Lu et al., 2016; Ayana et al., 2018; Kaur et al., 2018; Singh et al., 2018; Tomar et al., 2021). Kumar et al. (2008) also suggested that the resistance to SB is polygenic and controlled by a number of loci each having its own additive effect. This was further confirmed by Singh et al. (2018) in which lines were continuously distributed based on phenotypic screening, indicating that the resistance to leaf blight is certainly quantitative in nature. Latwal et al. (2016) reported that out of 200 wheat accessions obtained from CIMMYT, ∼5 lines were highly resistant, ∼123 lines resistant to moderately resistant, and 28 lines were susceptible to highly susceptible. A similar pattern was observed in the present study.

In the present study, five QTLs were mapped on chr 2A, 2B, 3B, 5B, and 6A. Three out of five QTLs (*QSb.pau-2A*, *QSb.pau-3B*, and *QSb.pau-5B*) had resistance allele donated by *A. speltoides* and are probably novel as no gene/QTL for SB resistance from *A. speltoides* has yet been reported, despite large genetic potential against SB (Smurova and Mikhailova, 2007). FBD, F-box, and Leucine Rich Repeat domains protein was 80.98 kb from the QTL *QSb.pau-2A*. NBS-LRR genes are the most common disease resistance gene family in plant genomes (Lee and Yeom, 2015; Dubey and Singh, 2018). A gene coding for ARF protein was found 5.68 kb from the QTL. As reported by Fu and Wang (2011), ARF regulates (enhance or repress) the transcription of primary auxin-responsive genes, thus involving auxin in biotic stress defense responses. Auxin-responsive genes are downregulated in *Arabidopsis thaliana* upon *Botrytis cinerea* infection making it more susceptible (Llorente et al., 2008). The genomic region of this SNP harbored another gene with Zinc finger CCCH domain-containing protein 32 (AtC3H32). Tandem CCCH zinc finger (TZF) motifs are known to play a variety of roles: ABA and gibberellin stress response (Lin et al., 2011), seed germination (Kim et al., 2008), mediated pathogen-associated molecular pattern (PAMP)–triggered immune responses (Maldonado-Bonilla et al., 2013), and involved in salt stress responses (Sun et al., 2007). Maldonado-Bonilla et al. (2013) reported that in *A. thaliana*, tandem zinc finger protein is phosphorylated by PAMP-responsive MAPKs which is required to trigger a PAMP-triggered immunity (PTI). Two genes coding for Cytochrome P450 were also found in the genomic region of the QTL and wheat Cytochrome P450 family protein is known to induce resistance to mycotoxin deoxynivalenol (DON) (Gunupuru et al., 2018). The cysteine protease coding gene in the region of the QTL also plays an important role as the extracellular cysteine protease is important for pathogen recognition. An oxidative burst is triggered by recognition, accompanied by transcriptional reprogramming and HR, which leads to disease resistance (Thomas and van der Hoorn, 2018). QTL *QSb.pau-2A* mapped in the present study was found 55 Mb from QTL *Q.Sb.bisa.2A* (Tomar et al., 2021) and in same genomic region of QTL *QSb.bhu-2A* (Kumar et al., 2010) and is probably novel as this QTL had been contributed by *A. speltoides* while previously reported QTL are from cultivated wheat.

QTL *QSb.pau-2B* was mapped 20 Mb from another QTL previously mapped in the region (Bainsla et al., 2020) and was found to be flanked by six senescence-associated family protein (DUF581) coding genes. In wheat, if one allele of the gene is involved in senescence, the other is associated with the stay-green trait (Tomar et al., 2021) and the stay-green trait has been reported to positively correlate with wheat leaf blight resistance (Joshi et al., 2006; Rosyara et al., 2008). QTL *QSb.pau-3B* was found to be linked with Protein FAR1-RELATED SEQUENCE 3 which is known to modulate plant immunity. FHY3 and its homolog FAR1 improve resistance by negatively regulating ROS accumulation and suppressing plant cell death (Ma and Li, 2018) and by positively regulating the biosynthesis of myo-inositol (Ma et al., 2016). The genomic region of the QTL was found to carry another gene coding for IQ domain-containing protein. Levy et al. (2005) reported that in *A. thaliana*, this protein, IQD1, encodes a novel nuclear protein that binds to calmodulin in a Ca^2+^-dependent fashion and stimulates accumulation of plant defense–related secondary metabolite glucosinolates. QTL *QSb.pau-3B* was mapped 28 Mb from QTL *QSb.bhu-3B* (Kumar et al., 2010) and hence this QTL introgressed from *A. speltoides* might be novel.

QTL *QSb.pau-5B* was found 20 Mb from earlier reported QTL *Q.Sb.bisa.5B* (Tomar et al., 2021) and 24 Mb from another QTL *S5B_679369233* (Jamil et al., 2018). Chromosome 5B has been reported as hotspot for SB resistance as a large number of QTLs/genes mapped for resistance against SB have been mapped on this chromosome. The annotation study revealed that the SNP S5B_703858864 linked to loci *QSb.pau-5B* is associated with three disease resistance protein coding genes. Both RP1 and AIG2 protein are known to play a crucial role in recognition of pathogens and effector-triggered immune responses in plants (Reuber and Ausubel, 1996; Beth Mudgett, 2005; Chisholm et al., 2006). The third resistance gene was NB-LRR gene which are the most common disease resistance gene family in plant genomes (Lee and Yeom, 2015; Dubey and Singh, 2018). The region also included six F-box family proteins. F-box family protein mediates a variety of biological processes, such as leaf senescence (Woo et al., 2001), and responses to biotic (Kim and Delaney, 2002) and abiotic stresses (Calderón-Villalobos et al., 2007). In mutant seedlings of *Arabidopsis* showing high susceptibility to pathogen *Peronospora parasitica*, Kim and Delaney (2002) have reported to isolate *son1* protein which was responsible to induce resistance among the seedlings. On cloning *son1*, it was found to encode a novel protein containing F-box motif, an element found within the E3 ubiquitin–ligase complex, suggesting the existence of a novel defense response through the ubiquitin–proteosome pathway, independent of SAR. The genomic region also carries gene encoding for ATP-dependent Clp protease ATP-binding subunit. Clp protease degrades damaged or non-native proteins in mitochondria and chloroplasts whose amount increases during abiotic and biotic stress conditions (Ali and Baek, 2020).

QTL *QSb.pau-6A* was mapped 53 Mb from QTL *SNP_3021829* (Bainsla et al., 2020) mapped in the same genomic region. A gene for Ubiquitin family protein was found flanking the QTL. Ubiquitin-related proteins implant plant resistance by degrading flagellin-sensing 2 (FLS2) receptor, which binds the microbe-associated molecular pattern (MAMP), flagellin (Trujillo and Shirasu, 2010; Lu et al., 2011). Ubiquitin, which is a part of the ubiquitin–proteasome system (UPS), controls various pathways including response to biotic and abiotic stresses (Sadanandom et al., 2012), and acts as one of the major systems in plant immunity (Üstün et al., 2016). Besides immunity, their role in defense responses by the production of ROS and forming hypersensitive reactions have also been reported (Marino et al., 2012). Another gene flanking the QTL coded for uricase. Increased activity of uricase has been observed in bean leaf tissue after infection with *Uromyces phaseoli* (Montalbini, 1991) in both resistant and susceptible plants. Higher activity of uricase was observed more in plants with hypersensitive reaction than in the susceptible plants.

The SNPs linked to QTLs were used to design KASP-based markers for marker-assisted transfer and validation. Using a susceptible high-yielding cultivar HD3086 and highly resistant DSBIL13, a BC_2_F_1_ population was generated. Since four of the five markers were not polymorphic between HD3086 and DSBIL13, only one marker S5B_703858864 linked to QTL *QSb.pau-5B* could be validated on the segregating population. The homozygous alternate alleles of this marker showed significant difference for SB severity with *p* value < 0.01, and thus this marker could be used for marker-assisted transfer of the QTL. The phenotypic evaluation of the segregating population showed a wide range of SB severity scores from highly resistant to highly susceptible, which indicated that more than one locus for resistance was segregating in the population. This segregation pattern was highly expected as DSBIL13 harbored four QTLs viz. *QSb.pau-2A*, *QSb.pau-2B*, *QSb.pau-3B*, and *QSb.pau-5B*. Thus, there is a need to explore more marker systems to design markers for marker-assisted transfer of other QTLs identified in the present study.

## Data Availability Statement

The datasets presented in this study can be found in online repositories. The names of the repository/repositories and accession number(s) can be found below: https://www.ncbi.nlm.nih.gov/bioproject/722517, PRJNA722517.

## Author Contributions

JKp and PC: design and supervision of the study. JKp, SK, AS, and PC: generation of plant genetic material. JKh, JKp, RB, HK, and JS: screening of material. JKh and GD: data analysis. JKh, JKp, RB, PS, SK, AS, and PC: management of trial. JKh, JKp, GD, and PC: draft of manuscript. All authors contributed for compilation of the article.

## Conflict of Interest

The authors declare that the research was conducted in the absence of any commercial or financial relationships that could be construed as a potential conflict of interest.

## Footnotes

1 https://www.lgcgroup.com
