# Supplementary material for: Characterization and Mapping of Spot Blotch in Triticum durum–Aegilops speltoides Introgression Lines Using SNP Markers
Source: Front Plant Sci. 2021 May 28;12:650400. doi: 10.3389/fpls.2021.650400 (PMC8193842; doi:10.3389/fpls.2021.650400)
Supplement: Supplementary file 1 [file Data_Sheet_1.docx]

**Characterization and mapping of spot blotch in *Triticum durum - Aegilopes speltoides* introgression lines using SNP markers**

**Jashanpreet Kaur^1^*, Jaspal Kaur^2^, Guriqbal Singh Dhillon^3^, Harmandeep Kaur^2^, Jasvir Singh^2^, Ritu Bala^2^, Puja Srivastava^2^, Satinder Kaur^3^, Achla Sharma^2^, Parveen Chhuneja^3^**

1. Department of Plant Pathology, Punjab Agricultural University, Ludhiana, India
2. Department of Plant Breeding and Genetics, Punjab Agricultural University, Ludhiana,
3. School of Agricultural Biotechnology, Punjab Agricultural University, Ludhiana, India

*Corresponding author email: [jashanpreet-coa@pau.edu](mailto:jashanpreet-coa@pau.edu)

Orcid ID

GSD: 0000-0001-6766-810X

SK: 0000-0003-3704-3074

PC: 0000-0002-8599-9479

JK: 0000-0002-5628-7325

Supplementary Tables

Table S1: Double digit scale (00-99) used for phenotyping germplasm against spot blotch of wheat (Eyal *et al* 1987)

| **Code** | **Scale for severity of infection** |
| --- | --- |
| **0** | No blight |
| **1** | Up to 10 % leaf area blighted |
| **2** | 11-20 % |
| **3** | 21-30 % |
| **4** | 31-40 % |
| **5** | 41-50 % |
| **6** | 51-60 % |
| **7** | 61-70 % |
| **8** | 71-80 % |
| **9** | >80 % leaf area blighted |

Table S2: Phenotypic evaluation for spot blotch disease severity of DSBILs along with recurrent parent (RP) and susceptible check across different environments

| **Env** | **Stage** | **RP** | **Check** | **Population** |  |  |  |  |  |  |
| --- | --- | --- | --- | --- | --- | --- | --- | --- | --- | --- |
|  |  | PDW274 | Raj4015 | Range | Median | Mean | Std.Dev | CV | Skewness | Kurtosis |
| BLUEs | FS | 14.50 | 38.00 | 0.25-39.00 | 11.25 | 12.71 | 8.42 | 0.66 | 0.96 | 0.54 |
|  | DS | 62.00 | 70.25 | 8.75-70.50 | 42.50 | 41.02 | 13.83 | 0.34 | -0.20 | -0.86 |
|  | HDS | 77.75 | 89.00 | 35.00-83.75 | 72.25 | 69.17 | 10.76 | 0.16 | -1.07 | 0.93 |
|  | AUDPC | 1081.25 | 1337.50 | 302.50-1228.75 | 828.75 | 819.55 | 213.24 | 0.26 | -0.23 | -0.73 |
| BLUPs | FS | 13.61 | 24.20 | 7.18-24.65 | 12.14 | 12.81 | 3.79 | 0.30 | 0.96 | 0.56 |
|  | DS | 52.92 | 57.58 | 22.82-57.73 | 41.90 | 41.09 | 7.76 | 0.19 | -0.21 | -0.83 |
|  | HDS | 75.00 | 82.81 | 45.32-79.17 | 71.18 | 69.06 | 7.43 | 0.11 | -1.09 | 1.01 |
|  | AUDPC | 985.25 | 1147.62 | 491.81-1078.71 | 825.26 | 819.86 | 134.52 | 0.16 | -0.24 | -0.70 |
| E1 | FS | 11 | 35 | 00-33 | 1 | 5.63 | 5.96 | 1.06 | 1.15 | 2.60 |
|  | DS | 55 | 57 | 00-68 | 24 | 28.99 | 17.45 | 0.60 | 0.54 | -0.64 |
|  | HDS | 55 | 89 | 00-89 | 47 | 43.75 | 20.83 | 0.48 | -0.40 | -0.93 |
|  | AUDPC | 880 | 1190 | 0-1175 | 530 | 536.80 | 269.56 | 0.50 | 0.32 | -0.49 |
| E2 | FS | 12 | 23 | 00-35 | 1 | 5.92 | 8.68 | 1.47 | 1.45 | 1.40 |
|  | DS | 67 | 78 | 11-78 | 35 | 37.28 | 20.39 | 0.55 | 0.36 | -0.94 |
|  | HDS | 89 | 99 | 37-89 | 89 | 83.74 | 8.23 | 0.10 | -2.44 | 9.73 |
|  | AUDPC | 1175 | 1390 | 445-1400 | 795 | 821.12 | 259.35 | 0.32 | 0.40 | -0.83 |
| E3 | FS | 13 | 37 | 00-48 | 12 | 21.25 | 21.27 | 1.00 | 1.36 | 0.95 |
|  | DS | 47 | 68 | 01-78 | 57 | 50.97 | 25.27 | 0.50 | -0.29 | -1.45 |
|  | HDS | 78 | 79 | 11-99 | 78 | 72.43 | 17.49 | 0.24 | -1.49 | 1.97 |
|  | AUDPC | 925 | 1260 | 135-1665 | 970 | 981.46 | 396.99 | 0.40 | -0.16 | -1.04 |
| E4 | FS | 22 | 57 | 00-57 | 13 | 17.99 | 15.14 | 0.84 | 0.88 | -0.10 |
|  | DS | 79 | 78 | 04-89 | 47 | 46.52 | 23.12 | 0.50 | -0.11 | -1.26 |
|  | HDS | 89 | 89 | 16-99 | 79 | 76.96 | 16.14 | 0.21 | -1.31 | 1.80 |
|  | AUDPC | 1345 | 1510 | 210-1655 | 980 | 941.52 | 357.82 | 0.38 | -0.11 | -0.95 |

# Standard deviation (Std.Dev), co-efficient of variation (CV), Skewness (Skew.), kurtosis (Kurt.), best linear unbiased predictions (BLUPs), best linear unbiased estimates (BLUEs), environment 1/season 2016-17 (E1), environment 2/season 2017-18 (E2), environment 3/season 2018-19 (E3), environment 3/season 2019-20 (E4), flowering stage (FS), dough stage (DS), hard dough stage (HDS), area under disease progression curve (AUDPC)

## Donor parent Aegilops speltoides (#pau3809) showed score 00 across all stages.

Table S3: QTLs along with SNPs and corresponding proteins and functional gene annotation elucidated based on the high confidence genes from wheat reference sequence (RefSeq V1.0) annotation database.

| **QTL** | **SNP** | **Chr** | **GeneID** | **Dist. (in kb)** | **Gene Annotation** |
| --- | --- | --- | --- | --- | --- |
| *Q.Sb.pau-2A* | S2A_755774702 | 2A | *TraesCS2A01G546600* | 439.059 | Cytochrome P450 family protein, expressed |
|  |  |  | *TraesCS2A01G546700* | 435.192 | Cysteine proteinase |
|  |  |  | *TraesCS2A01G546800* | 418.04 | Zinc finger MYM-type-like protein |
|  |  |  | *TraesCS2A01G546900* | 411.553 | Phytol kinase |
|  |  |  | *TraesCS2A01G547000* | 409.541 | Exostosin family protein |
|  |  |  | *TraesCS2A01G547100* | 317.21 | RNA-directed DNA polymerase (reverse transcriptase)-related family protein |
|  |  |  | *TraesCS2A01G547200* | 295.145 | ATP synthase subunit alpha |
|  |  |  | *TraesCS2A01G547300* | 95.506 | Polyadenylate-binding protein 1-B-binding protein |
|  |  |  | *TraesCS2A01G547400* | 81.978 | FBD, F-box and Leucine Rich Repeat domains containing protein |
|  |  |  | *TraesCS2A01G547500* | 71.745 | Serine/threonine-protein kinase |
|  |  |  | *TraesCS2A01G547600* | 37.854 | Cytochrome P450, putative |
|  |  |  | *TraesCS2A01G547700* | 33.721 | Peptidyl-tRNA hydrolase |
|  |  |  | *TraesCS2A01G547800* | 5.678 | Auxin response factor |
|  |  |  | *TraesCS2A01G547900* | -11.024 | Zinc finger CCCH domain-containing protein 32 |
|  |  |  | *TraesCS2A01G548000* | -15.993 | Phosphoribosylformylglycinamidine synthase subunit PurQ |
|  |  |  | *TraesCS2A01G548100* | -152.331 | Amine oxidase family protein |
|  |  |  | *TraesCS2A01G548200* | -199.456 | Amine oxidase family protein |
|  |  |  | *TraesCS2A01G548300* | -204.738 | Adenylate kinase |
|  |  |  | *TraesCS2A01G548400* | -225.895 | DUF594 family protein |
|  |  |  | *TraesCS2A01G548500* | -253.227 | Alpha-glucosidase |
|  |  |  | *TraesCS2A01G548600* | -282.781 | Alpha-glucosidase |
|  |  |  | *TraesCS2A01G548700* | -356.782 | Alpha-glucosidase |
|  |  |  | *TraesCS2A01G548800* | -390.864 | Gag-Pol polyprotein |
| *Q.Sb.pau-2B* | S2B_673595704 | 2B | *TraesCS2B01G476400* | 425.139 | senescence-associated family protein (DUF581) |
|  |  |  | *TraesCS2B01G476500* | 353.761 | senescence-associated family protein (DUF581) |
|  |  |  | *TraesCS2B01G476600* | -32.658 | senescence-associated family protein (DUF581) |
|  |  |  | *TraesCS2B01G476700* | -112.168 | senescence-associated family protein (DUF581) |
|  |  |  | *TraesCS2B01G476800* | -115.776 | senescence-associated family protein (DUF581) |
|  |  |  | *TraesCS2B01G476900* | -329.07 | senescence-associated family protein (DUF581) |
|  |  |  | *TraesCS2B01G477000* | -363.566 | zinc finger matrin-type protein |
|  |  |  | *TraesCS2B01G477100* | -431.149 | Glycosyltransferase |
|  |  |  | *TraesCS2B01G477200* | -434.456 | Nucleolar complex protein 4-like protein |
|  |  |  | *TraesCS2B01G477300* | -442.486 | Glutamate receptor |
| *Q.Sb.pau-3B* | S3B_104700839 | 3B | *TraesCS3B01G127000* | 375.807 | Protein FAR1-RELATED SEQUENCE 3 |
|  |  |  | *TraesCS3B01G127100* | -269.633 | IQ domain-containing protein |
| *Q.Sb.pau-5B* | S5B_703858864 | 5B | *TraesCS5B01G553200* | 370.394 | F-box family protein |
|  |  |  | *TraesCS5B01G553300* | 361.335 | F-box domain containing protein |
|  |  |  | *TraesCS5B01G553400* | 358.507 | F-box and associated interaction domains-containing protein |
|  |  |  | *TraesCS5B01G553500* | 329.318 | F-box domain containing protein, expressed |
|  |  |  | *TraesCS5B01G553600* | 304.847 | DNA topoisomerase |
|  |  |  | *TraesCS5B01G553700* | 214.322 | F-box family protein |
|  |  |  | *TraesCS5B01G553800* | 75.682 | Phytoene synthase, chloroplastic |
|  |  |  | *TraesCS5B01G553900* | 1.689 | F-box family protein |
|  |  |  | *TraesCS5B01G554000* | -214.708 | ATP-dependent Clp protease ATP-binding subunit |
|  |  |  | *TraesCS5B01G554100* | -232.54 | F-box family protein |
|  |  |  | *TraesCS5B01G554200* | -250.728 | Disease resistance protein RPM1 |
|  |  |  | *TraesCS5B01G554300* | -276.34 | Disease resistance protein (NBS-LRR class) family |
|  |  |  | *TraesCS5B01G554400* | -308.147 | Protein BCCIP homolog |
|  |  |  | *TraesCS5B01G554500* | -368.461 | AIG2-like (Avirulence induced gene) family protein |
|  |  |  | *TraesCS5B01G554600* | -380.912 | ADP-ribosylation factor GTPase-activating protein |
|  |  |  | *TraesCS5B01G554700* | -417.622 | 30S ribosomal protein S9 |
|  |  |  | *TraesCS5B01G554800* | -435.991 | Polynucleotidyl transferase, ribonuclease H-like superfamily protein |
|  |  |  | *TraesCS5B01G554900* | -442.454 | Protein kinase |
|  |  |  | *TraesCS5B01G555000* | -451.294 | Protein kinase |
| *Q.Sb.pau-6A* | S6A_131743987 | 6A | *TraesCS6A01G149400* | 300.685 | Tubby protein, putative |
|  |  |  | *TraesCS6A01G149500* | 297.87 | Ubiquitin family protein |
|  |  |  | *TraesCS6A01G149600* | -436.076 | Uricase |

# Distance from SNP (dist.) represent distance of start site of gene to SNP linked with QTL, where (+) sign represent that gene was found downstream of the SNP and (–) sign represent that gene was found upstream

Table S4: Primer sequences of SNP markers for validation in wheat lines by KASP assay.

| QTL | SNP | Allele Specific Primer-I (FAM) | Allele Specific Primer-II (HEX) | Common Primer |
| --- | --- | --- | --- | --- |
| *Q.Sb.pau-2A* | S2A_755774702 | GAAGGTGACCAAGTTCATGCT  agcttatcaagacacgttctagatt | GAAGGTCGGAGTCAACGGATT  agcttatcaagacacgttctagatc | tcaattaggcaccgggtacta |
| *Q.Sb.pau-2B* | S2B_673595704 | GAAGGTGACCAAGTTCATGCT  cagctcgacgctatctgctcc | GAAGGTCGGAGTCAACGGATT  cagctcgacgctatctgctct | ttcccggagtcttgatgcca |
| *Q.Sb.pau-3B* | S3B_104700872 | GAAGGTGACCAAGTTCATGCT  gtcaggccaggagacaca | GAAGGTCGGAGTCAACGGATT  gtcaggccaggagacacg | ctggtccgttccgttccc |
| *Q.Sb.pau-5B* | S5B_703858864 | GAAGGTGACCAAGTTCATGCT  gcttaggcacctcaccctc | GAAGGTCGGAGTCAACGGATT  gcttaggcacctcaccctg | cgatcttgagcgaggagtcc |
| *Q.Sb.pau-6A* | S6A_131743987 | GAAGGTGACCAAGTTCATGCT  ggtcgcgggttcgaAtcC | GAAGGTCGGAGTCAACGGATT  ggtcgcgggttcgaAtcT | aaaccgccattgagagggac |

Supplementary figures


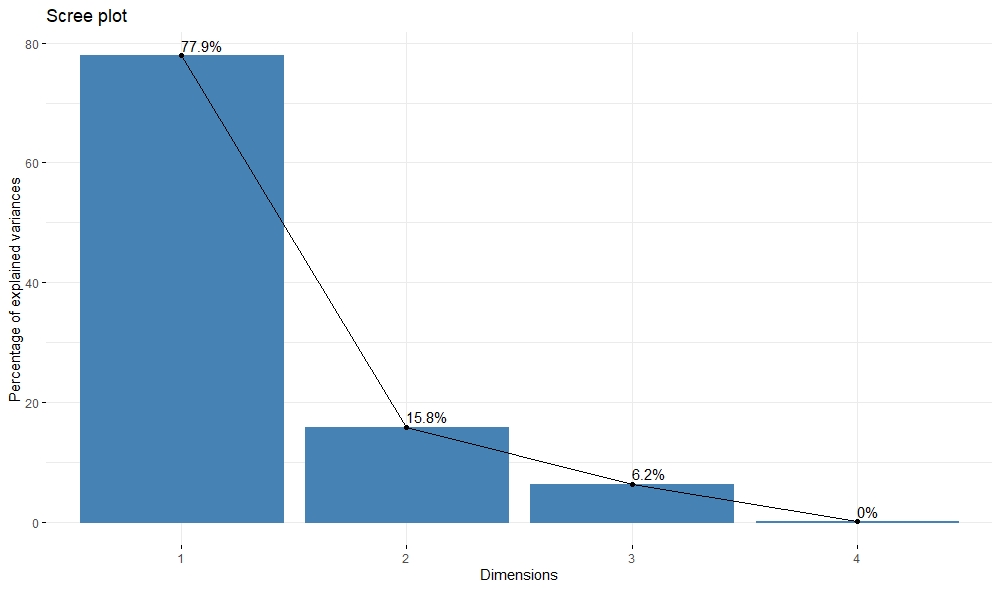


Figure S1: Scree plot for of percentage of variance explained by different dimensions of principal components.
